# How to Make It in the Urinary Tract: A Tutorial by *Escherichia coli*

**DOI:** 10.1371/journal.ppat.1002907

**Published:** 2012-10-04

**Authors:** Ine Jorgensen, Patrick C. Seed

**Affiliations:** 1 Department of Pediatrics, Duke University Medical Center, Durham, North Carolina, United States of America; 2 Department of Microbiology and Molecular Genetics, Duke University Medical Center, Durham, North Carolina, United States of America; University of North Carolina at Chapel Hill, United States of America

## Introduction

Extra-intestinal *E. coli* (ExPEC) may transition from benign colonization of the enteric and vaginal tracts to cause urinary tract infections (UTIs), septicemia, and meningitis. ExPEC colonization of the lower urinary tract leads to an acute infection of the superficial bladder urothelial cells, termed cystitis. Notably, 50% of all women will experience at least one UTI during their lifetime [1]. Even though ExPEC UTI induces a robust innate immune response, 25% of women with acute cystitis experience a second UTI within 6 months [2]. This failure to mount a protective immune response may be due to host genetic factors, antibiotic use, heterogeneity among ExPEC strains, active suppression of innate and adaptive immunity by ExPEC, and the ability of ExPEC to reside within protected, quiescent reservoirs. This review highlights the different stages of the ExPEC developmental cycle in the host bladder tissue, and describes the strategies employed by both players to gain the upper hand.

## Finding a Place to Hide: Attachment to and Invasion of the Bladder Epithelium

**Figure 1:**
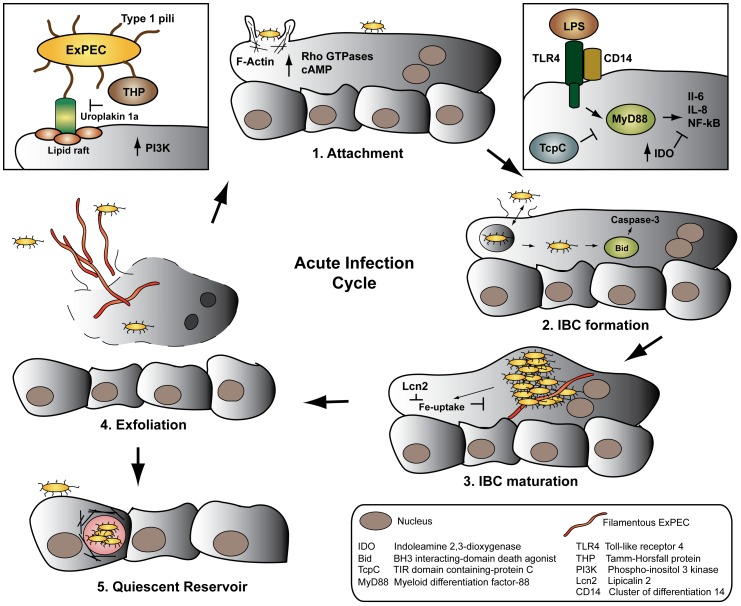
The ExPEC developmental cycle—a balancing act. ExPEC FimH binds uroplakin receptors on superficial bladder cells (1), initiating cytoskeletal rearrangements, invasion, and activation of the innate immune system. Following endosomal escape, ExPEC transitions from growth in bacterial intracellular communities (2–3), to filamentation (4), release from the host cell via exfoliation, and re-initiation of the infectious cycle. Occasionally, ExPEC establishes quiescent reservoirs in the underlying epithelium, serving as a reservoir for recurrent infections (5).

The urinary tract is a harsh physiological environment with constant urine flow, in which ExPEC must bind and invade the urothelial tissue. Of a wide range of cell surface-associated supramolecular adherence organelles expressed by ExPEC, P pili and type 1 pili (T1P) are well-established factors that mediate tissue tropism and adherence within the urinary tract. Additional ExPEC adhesins, such as S, Dr fimbrae, and Afa/Dr adhesins have been shown to facilitate adherence to urothelial cells in vitro, yet are not proven to play a major role in the pathogenesis of cystitis [3], [4]. T1P play a key role in murine cystitis: the T1P adhesin FimH binds mannosylated residues on the luminal bladder urothelial receptor uroplakin Ia associated with lipid rafts (Figure 1) [5]. Uroplakin Ia is considered the key host receptor, yet FimH also binds integrin, extracellular matrix proteins, and CD44. A large body of work has established T1P as a key virulence factor in mouse models of cystitis, but the exact role of T1P in human UTI remains debated: Inoculation of the non-human primate cynomolgus monkey with the T1P adhesin FimH yielded protection against ExPEC UTI [6], and children infected with T1P-expressing strains experience greater disease severity compared to children infected with T1P-negative isolates [7]. However, no difference in the level of inflammation was observed between a T1P-negative strain and a T1P-positive strain in a human-challenge model of UTI [8].

The ExPEC-urothelium interaction is further reinforced by ExPEC binding to the CD44 ligand hyaluronic acid (HA) in urine. The host counteracts T1P-mediated bacteria-bladder epithelium interaction by secreting renal-derived Tamm-Horsfall protein (THP) in urine. THP sequesters ExPEC from the epithelium by directly binding T1P via its mannose residues and thus blocks FimH binding to the uroplakin-receptor [9]. Intuitively, urine flow should aid in removing attached bacteria from the bladder surface. Instead, the sheer stress of urine flow strengthens FimH binding to mannose, locking the bacteria to the bladder surface [10].

Following attachment of ExPEC via T1P to the bladder surface, the bacteria are endocytosed in cyclic AMP (cAMP)-dependent Rab27b/CD63-positive vesicles [11]. As the bladder contracts and expels urine, low cAMP induces uptake of apical membranes in fusiform vesicles, in which ExPEC is passively internalized. Conversely, bladder-stretch and elevated cAMP signals Rab27-mediated exocytosis and expels fusiform vesicle-associated ExPEC [11], [12]. Internalization of ExPEC requires components of the host cytoskeleton: attachment to the plasma membrane induces activation of Rho GTPases, host tyrosine kinases, phosphoinositide-3-kinase, and focal adhesion kinase, followed by polymerization and re-organization of actin filaments at the site of attachment (Figure 1) [5]. The cytoskeletal proteins α-actinin and the two focal adhesion associated proteins tensin and talin further localize to the site of attachment, potentially stabilizing the rearranged actin. The histone deacetylase HDAC6 subsequently deacytelates α-tubulin, initiating a global rearrangement of microtubules [13]. Collectively, these signaling events mediate uptake by a zipper-like mechanism, reminiscent of other invading pathogens.

## Mounting an Army: IBC Formation

Whereas most internalized ExPEC are immediately cycled out of the urothelial cell by exocytosis [11], some bacteria escape and enter a cytosolic developmental stage called an intracellular bacterial community (IBC) (Figure 1). Initially, a single bacterium in the cytosol rapidly replicates in biofilm-like clusters of organisms. An IBC is not a membrane- or cytoskeletal-bound structure, but consists of an estimated 10^4^–10^5^ organisms in a highly organized bacterial community packed in a proteinaceous polysaccharide matrix. The IBC is so expansive that it causes the apical cell membrane to bulge into the bladder lumen (Figure 1) [9].

As the IBC matures, often enveloping the host nuclei, rod-like bacteria disperse from the periphery of the community, while other bacteria transition into long filaments that extrude from the infected cell (Figure 1). Both subpopulations may attach to adjacent epithelial cells and initiate another, less synchronous round of IBC formation [9]. T1P [14], outer membrane proteins [15], [16], and capsule [17] play essential roles in intracellular replication and IBC formation independent of their functions in attachment and invasion, underscoring the significant role of surface structures and biofilm-like factors in the ExPEC developmental cycle.

ExPEC alters the host immune response to infection through multiple mechanisms. ExPEC fails to filament in Toll-like receptor 4 (TLR4)-deficient mice, abrogating the development of a secondary round of IBC formation. Several models of murine cystitis demonstrate that the bladder innate immune response is primarily mediated by TLR4, resulting in NF-κΒ activation and IL-6 and IL-8 secretion [9]. Yet, ExPEC-infected urothelial cells are partially resistant to exogenous LPS- and TNF-α-induced cytokine secretion, indicating that ExPEC partially suppresses cytokine production in the bladder [5]. Recent evidence indicates that ExPEC further counteracts cytokine secretion by inducing expression of the anti-inflammatory enzyme Indoleamine 2,3-dioxygenase (IDO), which inhibits lymphocyte proliferation in response to cytokine secretion (Figure 1) [18].

## Hide and Seek: Establishment of a Quiescent Intracellular Reservoir

ExPEC assumes yet another developmental stage distinct from IBCs. As apical epithelial cells are continually shed into the urine by exfoliation, ExPEC may infect the underlying basal epithelium and establish a quiescent intracellular reservoir (QIR). Once internalized, the bacteria reside within LAMP-1-positive endosomes surrounded by a network of F-actin (Figure 1) [19]. In the absence of bacterial replication, the membrane-bound bacteria persist for months and are resistant to antibiotic treatment [20]. The mechanisms by which ExPEC remains latent, prevents engagement of the innate immune response, and re-initiates the infection are wholly unknown. Although the latent QIR population in any one animal is small, it is considered a major source of recurrent UTIs in women. Transitioning through the various stages of the developmental cycle, ExPEC traverses multiple bottlenecks – attachment, invasion, IBC, and QIR formation – which create clonal populations highly adapted to residing undetected within bladder tissue [9].

## The Host Fights Back

The host mounts a rapid and robust immune response to ExPEC colonization of the bladder, and severe inflammation is thought to predispose the bladder tissue to recurrent infections [21]. TLR4 cooperatively recognizes LPS and T1P in the bladder in a CD14-dependent manner, activating MyD88 and inducing downstream cytokine signaling [5]. Notably, the ExPEC protein TcpC binds MyD88 via its TIR-like domain, dampening downstream cytokine induction (Figure 1) [22]. Studies showing that TLR4^−/−^ mice maintain a high, persistent bacterial burden in the bladder [23] are in accordance with human data demonstrating that pediatric patients with congenitally low TLR4 expression on granulocytes are more likely to have prolonged asymptomatic bladder colonization [24]. Additionally, certain TLR4 promoter variants are associated with a reduced innate immune response to UTI in a human-challenge model [25]. Recent data, however, indicate that TLR4 is not the sole genetic determinant of susceptibility to UTI in mice, but rather a quantitative trait [26], [27]. Eventual clearance of the bladder infection is primarily mediated by IL-8-induced neutrophil recruitment to the infected tissue [9]. The ExPEC cytotoxic necrotizing factor 1 (CNF-1), however, interferes with neutrophil recruitment by modulating Rho GTPase activity important to neutrophil chemotaxis [28].

Exfoliation of infected superficial urothelial cells further aids in clearing the bladder infection. Caspase 3 is activated during the early stages of IBC formation, which leads to shedding of the infected urothelial cell, release of filamentous and rod-shaped ExPEC, and upregulation of bladder cell differentiation [5]. FimH-binding Uroplakin Ia is necessary and sufficient to induce Caspase 3 activation and shedding of the superficial bladder cells. Yet differentiation of superficial urothelial cells induced by Uroplakin III also plays an essential role in exfoliation: infected Uroplakin III-deficient urothelial cells in vitro fail to exfoliate, and undifferentiated infected bladder cells do not undergo apoptosis in a murine model of ExPEC bladder infection [29]. The ExPEC hemolysin HlyA further contributes to exfoliation by activating Caspase 3 and by promoting degradation of host proteins important for adherence [19]. Instinctively, induction of apoptosis by ExPEC factors aids in the clearance of the bladder infection. It is therefore interesting that ExPEC directly stabilizes the NF-κΒ activator IκΒ, inhibiting NF-κΒ-mediated upregulation of pro-survival, anti-apoptotic pathways [30]. It is conceivable that ExPEC strikes a balance where low levels of apoptosis are tolerated in the absence of a massive inflammatory response.

Iron sequestration is another strategy through which the host limits ExPEC growth. In a constant revolving battle, *E. coli* expresses iron-chelating siderophores such as Enterobactin, among other iron uptake systems, while the host expresses the iron-binding transferrin-like protein lactoferrin [5]. In response to TLR4 stimulation, the host further produces Lipocalin 2 (Lcn2), which directly inhibits *E. coli* siderophore function [31]. As countermeasures, ExPEC expresses several glycosyltransferases that render siderophores insensitive to Lcn2 (Figure 1) [32].

## Concluding Remarks and the Way Forward

ExPEC employs a myriad of complex, multifaceted strategies to combat the host immune response and to establish a niche in the harsh physiological environment of the bladder. Further investigation into the adaptive immune response to UTI and the establishment of quiescent reservoirs may reveal how and why some women experience recurrent UTIs, which remains the core clinical obstacle in treating ExPEC UTIs and a major socioeconomic burden. Since the majority of the data detailing the ExPEC developmental cycle during UTIs was elucidated in murine models, more work is required to fully understand the host-pathogen interaction in human patients.
